# Construction of professional identity in nursing students: qualitative research from the historical-cultural perspective*

**DOI:** 10.1590/1518-8345.3820.3284

**Published:** 2020-06-08

**Authors:** Rogério Silva Lima, Marta Angélica Iossi Silva, Luciane Sá de Andrade, Fernanda Dos Santos Nogueira De Góes, Maria Aparecida Mello, Marlene Fagundes Carvalho Gonçalves

**Affiliations:** 1 Universidade Federal de Alfenas, Escola de Enfermagem, Alfenas, MG, Brazil.; 2 Universidade de São Paulo, Escola de Enfermagem de Ribeirão Preto, PAHO/WHO Colaborating Centre at the Nursing Research Development, Ribeirão Preto, SP, Brazil; 3 Universidade Federal de São Carlos, Centro de Educação e Ciências Humanas, São Carlos, SP, Brazil.

**Keywords:** Students, Nursing, Education, Nursing, Baccalaureate, Nursing Education, Nursing Education Research, Social Learning, Human Development, Qualitative Research, Estudantes de Enfermagem, Bacharelado em Enfermagem, Pesquisa em Educação de Enfermagem, Aprendizado Social, Desenvolvimento Humano, Pesquisa Qualitativa, Estudiantes de Enfermería, Bachillerato en Enfermería, Investigación en Educación de Enfermería, Aprendizaje Social, Desarrollo Humano, Investigación Cualitativa

## Abstract

**Objective:**

to analyze the process of professional identity construction in undergraduate nursing students during their education.

**Method:**

qualitative research, anchored in the Historical-Cultural framework. Twenty-three undergraduate nursing students took part. Data were collected through individual interviews, with a semi-structured script. Thematic Analysis was used to analyze the data.

**Results:**

the following four themes were obtained, “The subject in movement to become a nurse: from previous experiences to entering the courses”; “The nursing professor in the construction of the undergraduate’s professional identity: a two-way mirror”; “Pedagogical relationship: instrument for constructing the student’s professional identity” and “Historical-cultural conditions: space for the construction of the student’s professional identity”.

**Conclusion:**

the construction of the students’ professional identity is limited to the material conditions of existence, translating appropriation to the intrapsychic scope of elements that occur, first, in the inter-psychological space of interactions. Nursing professors can become a paradoxical mirror, with one face to be imitated and the other, which materializes meanings of a model not to be followed. This construction is also influenced by the conditions of professional practice and university education.

## Abstract (pt)

**Objetivo:**

analisar o processo de construção da identidade profissional de enfermeiro pelos estudantes de graduação em Enfermagem no percurso de sua formação.

**Método:**

pesquisa qualitativa, ancorada no referencial Histórico-Cultural. Participaram 23 estudantes da graduação em Enfermagem. Os dados foram coletados por entrevistas individuais, com roteiro semiestruturado. Para análise dos dados, utilizou-se a Análise Temática.

**Resultados:**

obtiveram-se quatro temas, “O sujeito em movimento de tornar-se enfermeiro: das experiências prévias ao ingresso no curso”; “O professor enfermeiro na construção da identidade profissional do graduando: dois sentidos do mesmo espelho”; “Relação pedagógica: instrumento para construção da identidade profissional do aluno” e “Condições histórico-culturais: espaço de construção da identidade profissional do aluno”.

**Conclusão:**

a construção da identidade profissional dos alunos circunscreve-se às condições materiais de existência, traduz a apropriação para o âmbito intrapsíquico de elementos que se dão, primeiramente, no espaço interpsicológico das interações. Os professores enfermeiros podem se constituir espelho paradoxal, com uma face tomada para imitação, e outra que materializa significados de modelo a não ser seguido. Essa construção é também influenciada pelas condições do exercício profissional e da formação universitária.

## Abstract (es)

**Objetivo:**

analizar el proceso de construcción de la identidad profesional de los enfermeros por los estudiantes de pregrado de enfermería durante su formación profesional.

**Método:**

investigación cualitativa, anclada en el marco histórico-cultural. Participaron 23 estudiantes de pregrado de enfermería. Los datos fueron recolectados a través de entrevistas individuales, con un guion semiestructurado. Para el análisis de datos, se utilizó el análisis temático.

**Resultados:**

se obtuvieron cuatro temas, “El individuo en movimiento para convertirse en un profesional de enfermería: desde las experiencias previas hasta ingresar al curso”; “El maestro de enfermería en la construcción de la identidad profesional del estudiante: dos sentidos del mismo espejo”; “Relación pedagógica: instrumento para construir la identidad profesional del estudiante” y “Condiciones histórico-culturales: espacio para la construcción de la identidad profesional del estudiante”.

**Conclusión:**

la construcción de la identidad profesional de los estudiantes se limita a las condiciones materiales de existencia, traduciendo la apropiación al alcance intrapsíquico de elementos que ocurren, primero, en el espacio interpsicológico de interacciones. Los maestros de enfermería pueden convertirse en un espejo paradójico, con una frente que sirve de ejemplo, y la otra que materializa significados que indican un modelo que no se debe seguir. Esta construcción también está influenciada por las condiciones de la práctica profesional y la educación universitaria.

## Introduction

The term professional identity in Nursing is not always clearly grounded^(^ 1 ^)^. Perhaps because this field of study is marked by complexity, in close relationship with the diversity of theoretical frameworks supporting research^(^ 2 ^)^.

Nevertheless, nursing educators around the world share the concern that it is necessary to provide future nurses with space to construct professional identity^(^ 3 ^)^, especially in training, in order to provide conditions for the subject to appropriate cultural production aspects of what it means to be and to work as a nurse at a given historical and social moment. The construction of this identity is related, therefore, to the context of the profession, to the movements and activities of Nursing^(^ 4 ^)^.

In this sense, the dialectical perspective of human development - as a historical and cultural process, producing qualitative transformations, always in motion, enabling the subject to achieve self-determination^(^ 5 ^)^ - can contribute to understanding of the phenomenon.

Based on Historical-Cultural Theory, it is not possible to conceive of man, and therefore his identity, in a bipartite way and to study them in a dissociated way, that is, to seek to understand the psyche without behavior, or vice versa^(^ 6 ^)^, as studies would be reduced to explanatory-causal or comprehensive-descriptive readings.

Therefore, we seek to understand professional identity going beyond the dualism between the attributes inherent to the subject and the behavior expressed by a professional group. The professional identity of the nurse is conceived as a set of psychological functions integrated with each other, developed based on the multiple relationships of the subjects throughout their history and operated in the working relationships within the scope of work.

As eminently socio-historical functions, this development cannot be decontextualized from the conditions of its production, which raises the question: how are the cultural elements of the profession made available and how are they appropriated by students in the trajectory of constructing their professional nurse identity?

There is a need for research enabling understanding of the processes of constructing professional identity in nursing students so as to, on this basis, expand the possibilities of changing education^(^ 7 ^)^, sometimes still based on content paradigms, centered on the disease and on hospital practices^(^ 8 ^-^ 9 ^)^, in dissonance with what is expected of nurses nowadays.

Furthermore, the importance of such research emerges when one considers the historical moment that the nursing profession is going through, materialized in the worldwide Nursing Now campaign, which seeks to value and empower professionals in the face of health sector challenges^(^ 10 ^)^. It is understood that discussing the construction of professional identity in nursing students still in training can contribute important elements to achieving the results of this campaign, particularly in relation to the goal of improving education and professional development^(^ 11 ^)^. Especially because, the scope of the desired macro-political transformations presupposes a critical analysis of the dynamics of micropolitics, in the concrete spaces where the training of future nurses occurs^(^ 12 ^)^.

In view of these notes, this study aims to analyze the process of constructing professional identity in nurses by undergraduate nursing students during their education.

## Method

Qualitative research based on the Historical-Cultural framework, which guides the investigative effort in order to uncover the bases that are the genesis of a phenomenon, as the appearance and the way in which the phenomenon manifests depends on the conditions enabling its production^(^ 6 ^,^ 13 ^)^. Thus, an analysis is proposed that goes beyond the phenotypic surface, going beyond appearance.

The study was conducted with nursing students from a public university located in the southern region of the state of Minas Gerais (MG), Brazil. The study included: nursing students from the institution, in the eighth or ninth semesters of the degree, who agreed to participate in the study. This criterion was chosen because it is understood that at this stage students are in an important period of their training, in which they are expected to improve the skills required for professional practice, during internships. Those who had a professional qualification and/or experience as nursing technicians were excluded, as it is understood that the process of forming the professional identity of such subjects, as nurses, may follow another path involving professional or curricular experiences prior to the degree.

The data were collected between October 2016 and April 2017. During the period of data collection, 29 students were enrolled, of whom six had a professional qualification and/or experience as nursing technicians and were not included. A pilot study was previously carried out with four students to assess the relevance of the interview script. As it was not necessary to change the semi-structured script, the resulting data was included in the analysis. Therefore, 23 students participated in the research, selected by convenience. No one refused to participate.

The data were obtained by the main researcher through individual interviews, guided by a semi-structured script (Figure 1), the audio of which were recorded.

**Figure 1:** Semi-structured interview script. Minas Gerais, 2019

Guide questions	Topics investigated
1 – Why did you choose nursing a as profession?	The aspects of biography and cultural appropriations that circumscribed the professional choice and are characterized as conditions of possibility for constructing the professional identity of nurses.
2 – Why did you choose the nursing degree at this University?	
3 – In your opinion, what contribution does the nursing professor make to your professional education?	Interactions in learning spaces, particularly those with nursing professors, during the degree are fundamental elements in the trajectory to constructing professional identity.
4 - Has spending time with nursing professors during the degree influenced the formation of the nurse you intend to be? How? Please give examples.	
5 – During your professional education, did interaction with the nursing professor influence the way you view nursing as a profession? How? Please give examples.	
6 – How do you see yourself five years after completing your degree?	The close relationship between professional identity under construction and the material conditions of working as a nurse.
7 – Are there any questions you would ask yourself on this topic which were not ask during the interview? How would you respond?	Relevant aspects of the experiences from the students’ perspective which were not accessed by the previous questions

It is noteworthy that the interviewer researcher, although he did not share subjects with the participating students during the period, had already taught subjects throughout the undergraduate course, which encouraged approaching the students and the personal invitation to participate in the study. The interviews were conducted in a room at the university itself, with scheduling as requested by the respondents. The researcher and the interviewee remained alone in the environment in order to provide a welcoming atmosphere, without external interference.

To record the audio, a digital recorder was used. The total duration of the interviews was approximately six hours, varying between eight and 30 minutes each. The variation in time was related to the uniqueness of each participant in the way they expressed ideas, which did not represent a significant difference in the content of the interviews.

The interviews were transcribed in full in a text editor by the first author. The transcribed data totaled 101 pages.

Thematic analysis was used to analyze the data^(^ 14 ^-^ 15 ^)^, following six steps: familiarization with the data; generating initial codes, which was done inductively, based on the entire data set; elaboration of themes, that is, grouping a set of meanings related to ontological and epistemological assumptions that support the research construction; review of themes regarding external heterogeneity and internal homogeneity; defining and naming the themes; producing the research report.

The analysis was collaborative, carried out by the main researcher together with the research supervisor, with the critical contribution of the other authors^(^ 15 ^)^.

To ensure the rigor of the investigation, the procedures for collecting and analyzing data and the theoretical perspective that underpinned the research were explained, and the results were analyzed with interpretations in the light of the related literature^(^ 16 ^)^.

The research was conducted and the report produced in line with the criteria of the Consolidated criteria for reporting qualitative research^(^ 17 ^)^.

The study was approved by the Ethics and Research Committee (CEP), under opinion No. 1,691,082. The students signed an Informed Consent Form (ICF), after the purpose of the research had been explained to them. To preserve anonymity, the participants and teachers mentioned in the interviews were assigned fictitious names.

## Results

Thematic analysis of the data led to four themes, three of which had two subthemes (Figure 2).

**Figure 2:** Research themes and subthemes: The construction of professional identity in nursing students: qualitative research from the Historical-Cultural perspective. Minas Gerais, 2019

Themes	Subthemes
1 - The subject in movement to become a nurse: from previous experiences to entering the course	
2 – The nursing professor in the construction of the undergraduate’s professional identity: a two-way mirror	Professor: mirror of the nurse the student wishes to be
	Professor: mirror of the nurse the student does not wish to be
3 - Pedagogical relationship: instrument for constructing the student’s professional identity	Relationships leading to the recognition of the teacher as a model
	Relationships leading to suffering and crisis
4 - Historical-cultural conditions: space for the construction of the student’s professional identity	Nursing working conditions
	Higher education conditions in nursing

The first theme entitled “The subject in movement to become a nurse: from previous experiences to entering the course”, refers to participants’ mentions of their life history and the inter-influences of such elements on their choice of profession: [...] it was a passion that came from my father. He wanted to be a nurse, he did not take the course, so we always watched television programs about medical assistance, paramedics, and I liked it a lot. [...] When the end of high school arrived, I said - no, this is what I want! - and I chose nursing (Ana Maria, 23 years old, wanted to study nursing and intends to practice it).

The influence of the family was noted as a factor that awakens the choice of nursing as a career option, however, it was observed that it can also be a coercive choice: [...] I applied for several courses, several, I was accepted for civil engineering, [...] I was accepted for law [...] my brother is very much a reference for me like that, you know? Then he just said - you are going to do nursing! - I said - no, I won’t [...] You will! [...] If you don’t, you’re coming home tomorrow, I’ll pick you up! [...] (Joana, 25, wanted to study engineering and intends to practice nursing).

For some participants, nursing was not their first career choice:[...] In fact, I did not have in mind ... the plan to do nursing, I wanted to study medicine. When I saw that my score from the ENEM [National High School Exam] was not enough for medicine, I said - oh, so I’m going to apply for nursing - (Heloísa, 23 years old, wanted to study medicine and intends to practice nursing); [...] Because I always applied medicine, and I was not accepted for medicine, then I tried nursing [...] so, it is not what I really want in my life. My goal has always been medicine and it still is right? (Bruna, 37 years old, wanted to study medicine and does not intend to practice nursing).

However, our attention is attracted by the way the subject is reconfigured during the training. It can be seen, for example, that Heloísa changed her perception of the profession: [...] I have many colleagues who started nursing and really it was not what they had though and they wanted to switch to medicine, or even another profession. But I didn’t, I didn’t want to do nursing, but I started and now I love it and I wouldn’t swap it for medicine (Heloísa, 23, wanted to study medicine and intends to practice nursing).

The second theme, named “The nursing professor in the construction of the undergraduate’s professional identity: a two-way mirror”, shows the way teachers are perceived by students. Sometimes, the teacher is a model from which nursing takes shape for students, an aspect organized in the sub-theme “Professor: mirror of the nurse the student wishes to be”. [...] I could see, like, how important the competence of knowhow and such was, and then I kind of mirrored myself on that person, you know? On people, actually, because it wasn’t just one person. And then, we kind of mold ourselves [...] (Carla, 22, wanted to study biomedicine and intends to practice nursing); Ah, I think it’s important, because from the moment you see that your teacher is the professional - and we mirror our teachers - so the better the teacher the better the professional (Janaína, 25 years old, wanted to study medicine and intends to practice nursing).

On the other hand, the paradox that in the same work as nursing professor, particularly in the context of teaching professional practice in health services, students seem to delimit what they do not want to be. These meanings were organized in the sub-theme “Teacher: mirror of the nurse who does not want to be”. [...] there are teachers who say certain things in the classroom and then we arrive at the practice and see that they do not act as they should. [...] it is about humanization, ethical issues, issues of relationship even with other people. [...] There are people who speak, but don’t put it into practice (Denise, 22 years old, wanted to study physiotherapy and intends to practice nursing); [...] I used to say - guys, I never want to be a nurse like that, I’m going to leave this college, I’m not going back! [...] they only reinforce that I have to be better every day, that they are [...] incredible examples of how we must never be (Joana, 25, wanted to study engineering and intends to practice nursing).

The third theme, entitled “Pedagogical relationship: instrument for constructing the student’s professional identity” indicates that the teacher-student relationship in the teaching and learning processes is a tool that encourages the appropriation of meanings related to the nurses’ identity. In dissonance, other forms of interaction can distance students from this appropriation.

The subtheme “ Relationships leading to the recognition of the teacher as a model” shows recognition of the teacher as a support in the constitution of professional identity: [...] We discuss the case, the next day, we do the patient’s evolution, we take it home, we study, the next day comes, if I turn to the professor and talk say- I don’t know, I didn’t understand, I got home and researched but I didn’t understand, can you explain? - They explain, no problem (Lúcia, 23 years old, wanted to study nursing and intends to practice it).

Another component of the nursing professor’s work that seems to influence students’ identity lies in the management of contents and techniques in teaching the classes: He can teach, first he, let’s say it like this, he digests all this, and then he knows how to explain it to us in a way that we know how to understand [...] (Camila, 25, wanted to study nursing and intends to practice it).

The other side of the act of teaching manifests interactions that can generate crises, as shown in the subtopic “Relationships leading to suffering and crisis”. *I think after I started college.* [...] I became a totally anxious person, I discovered health problems ... [...] because of college! (Researcher) - Do you attribute this to the undergraduate course? - (Lúcia) To the teachers (Lúcia, 23 years old, wanted to study nursing and intends to practice it); [...] Ah, don’t put too much pressure as the teachers put it [...] then we even go to the practicals scared. [...] Sometimes you are even afraid to say something and be wrong, and have the professor point it out, you know? Lose marks (Elaine, 26, wanted to study physiotherapy and intends to practice nursing).

The way in which the teacher acts in the classroom can pose obstacles to learning and distance students from appropriating the meanings related to professional identity: [...] we end up discussing, talking like this - wow, but how, what about the didactics of this professor ... when he arrives to teach, it seems that he does it under duress, you know? [...] arrives, read the slide [...] I go, get the book, and read it (Lucas, 21 years old, wanted to study health and intends to practice nursing).

Regarding the fourth and last theme, called “Historical-cultural conditions: space for the construction of the student’s professional identity”, it was found that the socially constituted conditions of professional practice, as well as the conditions in which training takes place, translated into conformation of the curricular structure, playing a role in the configuration of professional identity.

In the subtheme “Nursing working conditions”, the way in which socially elaborated meanings about the profession accompany students in their trajectory is presented: [...] people say ... [...] - wow, but you are going to do nursing, wow, but it is such a bad course, so undervalued, you earn so little - [...] The general population [...] Nobody congratulates you [...] (Roberto, 24 years, wanted to study nursing and intends to practice it).

Throughout training and approaching the end of the course, students are faced with this question: Ah, it is because throughout the entire degree, it seems that I only came across professionals who were not happy, speaking ill of the profession, speaking of lack of recognition, saying they earn little (Janaína, 25 years old, wanted to study medicine and intends to practice nursing).

From the students’ perspective, training, as it is structured, can also pose some obstacles to professional development. This aspect of the results was organized in the subtheme “Higher education conditions in nursing”.

The students pointed out factors that, in their view, favor professional training. [...] we could have gone more in depth with the teacher there, especially now at the end, we don’t spend much time with the teacher there. [...] I was going to be able to have other experiences [...] this could give a little more feeling of security [...] (Roberto, 24, wanted to study nursing and intends to practice it); [...] they could try to expand this curriculum a little ... this practice time, you know? [...] instead of just learning the theoretical part (Camila, 25, wanted to study nursing and intends to practice it).

Moreover, clinical experience is not always dialogical and inclusion in health care services during the course is sometimes restricted to observation or does not provide clarity about what it means to be a nurse: [...] because we aren’t able to debate right? To say - No, you’re wrong, you don’t have to do it that way, because we’re still students, so we have more to watch and learn than to answer, right? (Luana, 23, wanted to study nursing and intends to practice it); [...] this leadership business left a lot to be desired, sometimes I feel like a leader, other times I don’t feel like a leader [...] Sometimes those who talk a lot [...] in the classroom and in practice it’s a little ... abstract [...] (Vivian, 23 years old, wanted to study nursing and intends to practice it).

## Discussion

The trajectory of constructing the nursing student’s professional identity is related to their life history and circumscribed to the material conditions of existence from which it is constituted, through the appropriation of the cultural elements available to them.

Students go into nursing due to the historical possibilities that circumscribe them. For example, for some participants, access to university was through the nursing course - in view of lower competition, compared to other courses in the health care field^(^ 18 ^)^. Although the first course option for some students was medicine, they were unable to access it, restricted, in most cases, to a group of students due to the competition^(^ 19 ^)^.

For the nursing workforce, this is particularly important. If, historically, medicine tends to be the first choice of students who aspire to careers in the health care field^(^ 20 ^)^, nursing can bring together students who do not wish to become nurses and choosing to enter the profession without awareness of its duties^(^ 21 ^)^, which can impact how it is valued by the student him/herself and the future professional of the area.

Thus, incentivizing policies must be put in place to recover the historical and cultural value of the nurse’s profession, strengthening social and political positioning as central aspects to the health and development of society^(^ 10 ^)^.

Nevertheless, the students’ statements enable us to infer that the subjects can reframe the profession. Although it cannot be guaranteed that completion of the course will result in effective professional practice, the fact that a student enters the course without consciously wanting it and concludes it by reconfiguring thinking, highlights the importance of the training process.

This is related to the assumption that the learning process leads to the continuous development of higher psychological functions and education not only influences the development processes in isolation, but can restructure the subject’s functions in all its breadth^(^ 13 ^)^.

Based on new needs, human beings create a different appropriation of the meanings of the objects around them. Consequently, they dialectically transform it, to the same extent that they modify their own surroundings^(^ 6 ^)^. Thus, the learning that occurs during the degree may modify the student’s development in other fields of life, with consequent new formations of personal purposes and world view.

In continuous interaction with social actors throughout their education, students can develop resources and functions to adapt to the requirements of the prospective career^(^ 22 ^)^ or even rethink their choices.

In this process, the role of teachers, as nurses, seems important for students to materialize what it means to be and work as a nurse. The professors’ role in this construction is not limited to the classroom^(^ 23 ^)^ and their contextualized performance in the fields of practice occupied a prominent place in this study.

The path to constructing professional identity as nurses, by students, may be similar to the trajectory formulated in the Cultural Development Law, which assumes that every higher psychological function is rather a socio-cultural formation, beyond the biological field^(^ 6 ^)^. Thus, at first, being a nurse happens in the interpsychological sphere, among peers. The professor, as a nurse, demonstrates conduct, skills, materializes attributes and indicates meanings, and based on this relationship, the students appropriates what, in itself, it means to be a nurse, presenting himself as a nurse to the other, professor and peers, and, at the same time, becoming a nurse for themselves.

Based on what they demonstrate by the way in which they operate the different characteristics of being a nurse, professors are a model for the student^(^ 3 ^,^ 24 ^)^.

Professional models are perceived as those who influence others, exemplifying ways of acting, professional and/or personal characteristics expected in nursing, and which can be imitated by others^(^ 25 ^)^. Imitation, in turn, is a highly important mechanism in the transformations which occur in personality functions, because it is a process that allows learning to happen in collaboration with the other, leading to development and transitions^(^ 13 ^)^.

This aspect is noted in the interviews, mainly regarding interactions between professors and students in clinical practice environments. There is a consensus in the literature that clinical practice is an essential part of nursing education^(^ 26 ^)^, it gives meaning to theory^(^ 3 ^)^ and doing linked to practice is central for the student to develop professional identity as a future nurse^(^ 27 ^-^ 28 ^)^. In line with this finding, studies have observed that the manifestation of values by professional models, in the practical field, is a great incentive for students to develop these characteristics^(^ 29 ^)^. Students can take teachers as examples and seek to appropriate professional attributes at the beginning of training, during the internship, and in the future career^(^ 23 ^)^.

On the other hand, it was observed that models perceived by students as inadequate also play a role in the process of developing professional identity. There are authors who infer that students can even learn from professional models whose attitudes are not consistent with their practice^(^ 25 ^)^ and that professors’ perceived lack of empathy pushes nursing students to work towards developing a better standard than that perceived in their professors^(^ 30 ^)^.

However, interacting with examples with inconsistent attitudes can cause loss of interest in learning, feeling that you will not be able to be a successful nurse^(^ 30 ^)^, difficulties in developing personal strategies to avoid becoming the same as the models you criticize^(^ 31 ^)^ and appropriation of inappropriate conduct that may be replicated in professional practice^(^ 32 ^)^.

These points, combined with the conception of learning as a process, dependent on the collaboration of others, which aims to provide the individual with mastery over what cannot be done alone^(^ 13 ^)^, highlight the role of the nursing professor in the students’ process of constructing identity.

Relationships established during the degree play a role in this dynamic, given that the student’s education is not restricted to the formal curriculum, but takes into account subjective aspects^(^ 32 ^)^ that also impact learning and professional development^(^ 33 ^-^ 34 ^)^.

From this angle, an empathic relationship can lead to a constructive learning experience, and thus, students feel interested, motivated to learn more, continue their studies and achieve better results^(^ 30 ^)^. Establishing such a relationship is facilitated by mutual trust, understanding, care and clear guidelines^(^ 33 ^)^.

However, verticalized relationships may result in distancing students from spending time with possibly significant models for constituting identity. Some professors’ attitudes, such as criticism unaccompanied by encouragement and reproving errors, can distance students and generate feelings such as fear of asking questions^(^ 33 ^-^ 34 ^)^, looking stupid, not knowing anything^(^ 30 ^)^, in addition to anxiety about attitude to evaluation and inappropriate communication from the instructor^(^ 35 ^)^.

Another important aspect in the findings of this study concerns the influences of sociocultural constructs on nurses’ professional practice.

Despite changes in the last decade, perceptions that undervalue nursing and careers in the field sometimes persist in the social imagery^(^ 36 ^)^. Stereotypes expressed in the media and low financial remuneration given to significant nursing segments in Brazil, raise doubts about nursing as a choice of profession^(^ 4 ^)^. Such perceptions influence some of the aspirants to the profession^(^ 36 ^)^.

This devaluation is also expressed in precarious work relationships, with an unstable labor market, characterized by fixed-term employment contracts^(^ 37 ^)^, double shifts and elevated weekly workload^(^ 38 ^)^.

It is recognized that students need to support their decisions to overcome the difficulties they encounter throughout their training when faced with these objectives and subjective elements. Therefore, we need to recognize the contradictions permeating the professional practice of nurses these days.

We can infer the need for the student to have experiences which manifest the, sometimes unfavorable, material conditions, of the nurse’s professional practice. However, special care must be taken with the type of interaction established, as it may or may not provide understanding and dialogue about the dynamics maintaining these conditions and provide space for critical analysis of culturally established contradictory elements, manifesting themselves in organizational hierarchies, in historical consolidation and in legal and educational aspects of the profession^(^ 39 ^)^.

Also highlighted in this investigation was the imbricated influence of the training structure, through curriculum dynamics, in constructing students’ professional identity. Students point out that the restricted practical workload, in addition to other factors such as the internship supervision model, disadvantage their professional development.

If clinical experience in health care services is essential for students to build their professional identity^(^ 28 ^)^, if there is only a short time in these spaces, learning may be disadvantaged and not provide appropriation of the founding elements of the profession^(^ 26 ^)^.

Likewise, depending on how student supervision takes place, they will not always be systematically accompanied by a professor linked to the course and, in theory, with training to articulate pedagogical and specific knowledge.

Although national curricular guidelines prescribe that nurses in health care services where internships take place must effectively participate in preparing and supervising students^(^ 40 ^)^, multiple socioeconomic and political factors stand in the way of achieving this objective.

Furthermore, health care services in which clinical practice occurs during the training period may have limited governance^(^ 41 ^)^ and hierarchical relationships^(^ 26 ^)^ delimiting the teaching possibilities materialized in the curricular proposals.

Thus, what is desired to be operationalized in training is not always possible, given the material conditions circumscribing educational proposals, such as the hegemony of the disciplinary and traditional teaching model, the difficulties in the partnership between teaching and service and the way of hiring nursing professors^(^ 42 ^)^.

Moreover, it is necessary to recognize that working nurses do not always have the preparation necessary to support students’ learning^(^ 26 ^,^ 43 ^)^. Thus, it is understood that the way training is structured and learning is provided, which professors and students may not pay attention to^(^ 44 ^)^, is an important element in the trajectory of constructing identity.

The results of this research are corroborated by national and international literature, which highlights the global concern with the training processes in nursing, the role of professors, the curriculum and its consequences in producing the nurses of the future and in the way the profession is valued.

This research has some limitations, such as the fact that it was conducted in only one educational institution, which translates specific socio-cultural conditions, and the data were produced from interviews alone. However, when privileging the interviews, it was considered that it is mainly through language that the system of historically constructed meanings and the meanings singularly attributed by the students are accessed^(^ 13 ^)^, elements essential to understanding the complex learning processes related to professional identity.

From this understanding, the analysis presented, based on a theoretical framework^(^ 6 ^,^ 13 ^)^ not commonly used to approach the topic, have enabled advances in this field of studies to the extent that we can have a different view of interaction as a psychological tool that may or may not favor the appropriation of the meanings in the nurse’s identity.

This study can contribute to curricular changes by reinforcing the importance of providing appropriate spaces, quantitatively and qualitatively, for establishing interactions that enhance the development of professional identity, which implies establishing strategies to improve teaching-service partnerships and adopting positioning contrary to distance-learning nursing courses.

## Conclusion

The construction of the students’ professional identity is influenced by their life trajectory and limited to the material conditions of each subject’s existence. It follows a path that translates appropriation to the intrapsychic scope of elements that take place, primarily, in the interpsychological space, and are apparent from interactions, especially those made possible by training, with emphasis on those between nursing professors and students.

For students, the nursing professor can become a paradoxical mirror, with one face imitation, and the other materializing meanings indicating a model not to be followed.

This process of constructing professional identity is not detached from the conditions that recursively result in the configuration of the nurse’s professional practice and the professional training itself.

## References

[1] Johnson M, Cowin LS, Wilson I, Young H (2012). Professional identity and nursing: contemporary theoretical
developments and future research challenges. Int Nurs Rev.

[2] Cardoso I, Batista P, Graça A (2014). Professional identity in analysis: a systematic review of the
literature. Open Sports Sci J.

[3] Marañón AA, Pera MPI (2015). Theory and practice in the construction of professional identity
in nursing students: a qualitative study. Nurse Educ Today.

[4] Silva AR, Padilha MI, Backes VMS, Carvalho JB (2018). Professional nursing identity: a perspective through the
Brazilian printed media lenses. Esc Anna Nery.

[5] García LD (2015). El desarrollo psicológico humano como processo de continuidade y
ruptura: La "Situacíon social del desarrollo". Educ Filos.

[6] Vygotski LS (2000). Obras escogidas III: problemas del desarrollo de la psique.

[7] Baldwin A, Mills J, Birks M, Budden L (2014). Role modeling in undergraduate nursing education: an integrative
literature review. Nurse Educ Today.

[8] Cassiani SHB, Wilson LL, Mikael SSE, Peña LM, Grajales RAZ, McCreary LL (2017). The situation of nursing education in Latin America and the
Caribbean towards universal health. Rev. Latino-Am. Enfermagem.

[9] Fortuna CM, Matumoto S, Mishima SM, Rodríguez AMMM (2019). Collective health nursing: desires and practices. Rev Bras Enferm.

[10] Kennedy A (2019). Wherever in the world you find nurses, you will find
leaders. Rev. Latino-Am. Enfermagem.

[11] Cassiani SHB, Lira JCG (2018). Nursing perspectives and the "Nursing Now"
campaign. Rev Bras Enferm.

[12] Chaves SE (2014). Macropolitical and micropolitical movements in the undergraduate
teaching on nursing. Interface.

[13] Vygotski LS (2001). Obras escogidas II: problemas de psicología general.

[14] Braun V, Clarke V (2006). Using thematic analysis in psychology. Qual Res Psychol.

[15] Braun V, Clarke V (2019). Reflecting on reflexive thematic analysis. Qual Res Sport Exerc Health.

[16] Ryan F, Coughlan M, Cronin P (2007). Step-by-step guide to critiquing research. Part 2: Qualitative
research. Br J Nurs.

[17] Tong A, Sainsbury P, Craig J (2007). Consolidated criteria for reporting qualitative research (COREQ):
a 32-item checklist for interviews and focus groups. Int J Qual Health Care.

[18] Puertas Cañaveral IC, Sá OAT (2017). REUNI: expansion, segmentation and institucional determination of
dropout. Case study at Unifal-MG. Eccos.

[19] Wickbold CC, Siqueira V (2018). Quotas policy, curriculum and identity construction of medical
students at a public university. Pro-Posições.

[20] Teodosio SS, Padilha MI (2016). To be a nurse: a professional choice and the construction of
identity processes in the 1970s. Rev Bras Enferm.

[21] Barbarà-i-Molinero A, Cascón-Pereira R, Hernández-Lara AB (2017). Professional identity development in higher education:
influencing factors. IJEM.

[22] Porteous D, Machin A (2018). The lived experience of first year undergraduate student nurses:
a hermeneutic phenomenological study. Nurse Educ Today.

[23] Baldwin A, Mills J, Birks M, Budden L (2017). Reconciling professional identity: a grounded theory of nurse
academics' role modelling for undergraduate students. Nurse Educ Today.

[24] González-Aguilar  A, Vázquez-Catano F, Almazán-Tlalpan, Morales-Nieto, Garcia-Solano (2018). Process of professional identity perception in
nursing. Rev Cuidarte.

[25] Felstead IS, Springett K (2016). An exploration of role model influence on adult nursing students'
professional development: a phenomenological research study. Nurse Educ Today.

[26] Lee JJ, Clarke CL, Carson MN (2018). Nursing students' learning dynamics and influencing factors in
clinical contexts. Nurse Educ Pract.

[27] Williams MG, Burke LL (2015). Doing learning knowing speaking: how beginning nursing students
develop their identity as nurses. Nurs Educ Perspect.

[28] Ewertsson M, Bagga-Gupta S, Allvin R, Blomberg K (2017). Tensions in learning professional identities - nursing students'
narratives and participation in practical skills during their clinical
practice: an ethnographic study. BMC Nurs.

[29] Shafakhah M, Molazem Z, Khademi M, Sharif F (2016). Facilitators and inhibitors in developing professional values in
nursing students. Nurs Ethics.

[30] Mikkonen K, Kyngäs H, Kääriäinen M (2015). Nursing students' experiences of the empathy of their teachers: a
qualitative study. Adv Health Sci Educ Theory Pract.

[31] Traynor M, Buus N (2016). Professional identity in nursing: UK students' explanations for
poor standards of care. Soc Sci Med.

[32] Felstead I (2013). Role modelling and students' professional
development. Br J Nurs.

[33] Chan ZCY, Tong CW, Henderson S (2017). Power dynamics in the student-teacher relationship in clinical
settings. Nurse Educ Today.

[34] Chan ZCY, Tong CW, Henderson S (2017). Uncovering nursing students' views of their relationship with
educators in a university context: a descriptive qualitative
study. Nurse Educ Today.

[35] Arkan B, Ordin Y, Yilmaz D (2018). Undergraduate nursing students' experience related to their
clinical learning environment and factors affecting to their clinical
learning process. Nurse Educ Pract.

[36] Girvin J, Jackson D, Hutchinson M (2016). Contemporary public perceptions of nursing: a systematic review
and narrative synthesis of the international research
evidence. J Nurs Manag.

[37] Oliveira JSA, Pires DEP, Alvarez AM, Sena RR, Medeiros SM, Andrade SR (2018). Trends in the job market of nurses in the view of
managers. Rev Bras Enferm.

[38] Oliveira BLCA, Silva AM, Lima SF (2018). Weekly workload for nurses in Brazil: challenges to practice
profession. Trab Educ Saúde.

[39] Strouse SM, Nickerson CJ (2016). Professional culture brokers: nursing faculty perceptions of
nursing culture and their role in student formation. Nurse Educ Pract.

[40] Resolução CNE/CES n. 3 de 07 de novembro de 2001 (BR) (2001). Institui Diretrizes Curriculares Nacionais do Curso de Graduação
em Enfermagem. Diário Oficial da União.

[41] Lima MM, Reibnitz KS, Kloh D, Silva KL, Ferraz F (2018). The pedagogical relationship in practical-reflexive education:
characteristic elements of teaching integrality in nurse
education. Texto Contexto Enferm.

[42] Soriano ECI, Peres CRFB, Marin MJS, Tonhom SFR (2015). State nursing courses in São Paulo foward the curriculum
guidelines. REME.

[43] Hanson SE, Macleod ML, Schiller CJ (2018). It's complicated: staff nurse perceptions of their influence on
nursing students' learning. A qualitative descriptive study. Nurse Educ Today.

[44] Liljedahl M, Boman LE, Fält CP, Laksov KB (2015). What students really learn: contrasting medical and nursing
students'experiences of the clinical learning environment. Adv Health Sci Educ Theory Pract.

